# *Calea zacatechichi* dichloromethane extract exhibits antidiarrheal and antinociceptive effects in mouse models mimicking irritable bowel syndrome

**DOI:** 10.1007/s00210-015-1142-1

**Published:** 2015-06-12

**Authors:** M. Sałaga, A. Kowalczuk, M. Zielinska, A. Błażewicz, J. Fichna

**Affiliations:** Department of Biochemistry, Faculty of Medicine, Medical University of Lodz, Mazowiecka 6/8, 92-215 Lodz, Poland; National Medicines Institute, Warsaw, Poland

**Keywords:** Diarrhea, Abdominal pain, Gastrointestinal motility

## Abstract

*Calea zacatechichi* Schltdl. (Asteraceae alt. Compositae) is a Mexican plant commonly used in folk medicine to treat respiratory and gastrointestinal (GI) disorders. The objective of this study is to characterize the effect of *C. zacatechichi* extracts in mouse models mimicking the symptoms of irritable bowel syndrome (IBS). Powdered *C. zacatechichi* herb (leaves, stems, and flowers) was extracted with methanol. Methanolic extract was filtered and evaporated giving methanolic fraction. The residue was extracted with dichloromethane (DCM). Methanolic and DCM (200 mg/kg, per os) extracts were screened for their effect on GI motility in several in vitro tests, and the antidiarrheal and antinociceptive effects were assessed using mouse models. The influence of the DCM extract on motoric parameters and exploratory behaviors was also assessed. Finally, the composition of *C. zacatechichi* DCM extract was qualitatively analyzed using liquid chromatography-mass spectrometry (LC-MS) method. *C. zacatechichi* DCM extract significantly inhibited the contractility of mouse colon in vitro (IC_50_ = 17 ± 2 μg/ml)*.* Administration of the DCM extract in vivo (200 mg/kg, per os) significantly prolonged the time of whole GI transit (46 ± 1 vs. 117 ± 27 min for control and DCM-treated animals, respectively; *P* = 0.0023), inhibited hypermotility, and reduced pain in mouse models mimicking functional GI disorders. Our findings suggest that constituents of the *C. zacatechichi* DCM extract exhibit antidiarrheal and analgesic activity. The extract may thus become an attractive material for isolation of compounds that may be used as a supplementary treatment for pain and diarrhea associated with IBS in the future.

## Introduction

Irritable bowel syndrome (IBS) is a functional gastrointestinal (GI) disorder with an estimated prevalence of 10–20 % (Fichna and Storr 2012). According to Thompson et al. (2000), it accounts for about 3 % of all general practice and up to 40 % of all GI referrals. Symptoms of IBS, which include abdominal pain, altered stool consistency and frequency, diarrhea, and/or constipation, are caused mainly by disturbances in intestinal motility and/or secretion. Although IBS is not life-threatening, it is a heavy economic burden due to increased work absenteeism and impaired quality of life of its sufferers, as well as increased use of health care services (Sandler et al. 2002; Fichna and Storr 2012). Current understanding of the pathogenesis of IBS is unsatisfactory due to the lack of demonstrable pathological abnormalities and reliable biomarkers (Fichna and Storr 2012). There are several hypotheses concerning the pathology of IBS. One of them, which is based on the analyses of specimens obtained at endoscopy and in serological cytokine studies, shows IBS as a localized low-grade inflammatory disorder with mast cells (MC) playing a particularly important role (Mayer and Collins 2002; Philpott et al. 2011; Fichna and Storr 2012). An alternative hypothesis states that food allergy may play a key role (Atkinson et al. 2004). Concurrently, the bi-directional communication between the gut and the central nervous system (CNS) often related to as the brain-gut axis (BGA) is thought to have a crucial role in IBS pathogenesis (Fichna and Storr 2012).

At present, pharmacological therapy for IBS focuses on alleviation of its symptoms, such as diarrhea or abdominal pain, but not on the removal of its cause, which is mostly unrecognized. There are several conventional and complementary therapies, as well as herbal remedies for IBS, which have been recently summarized by Yoon et al. (2011) and Rahimi and Abdollahi (2012). *Calea zacatechichi* Schltdl. (Asteraceae alt. Compositae) is a Mexican plant also known as Dream Herb or, according to the Indian word “zacatechichi,” Bitter Grass. It is native to Mexico and Central America. *C*. *zacatechichi* has been used for centuries by Chontal Indians for rituals aiming at dream-based divination, what suggests CNS-mediated, hallucinogenic action (Wu et al. 2011). Moreover, some Indian tribes such as Zoque Popoluca have been using *C. zacatechichi* as a remedy for cough and asthma, as well as GI tract disorders, such as stomachache and diarrhea (Leonti et al. 2003). To date, several biologically active compounds were isolated from the plant. Recently, Wu et al. (2011) reported presence of six germacranolides, which were shown to have antileishmanial activity. Bork et al. (1997) found that the ethanolic extract from *C. zacatechichi* leaves contains biologically active sesquiterpene lactones, which were shown in vitro to inhibit activation of NF-κB, a transcriptional factor and one of the major mediators of inflammatory pathways. Other compounds and their biological actions such as calaxin, ciliarin, caleins A and B, caleicins I and II, acactein, zexbrevin, and neurolenin B were all reported earlier in papers ranging from 1970 to 1980 (Wu et al. 2011).

In our search for possible novel treatments for functional GI disorders, we employed two different extracts from *C. zacatechichi* and tested their effect on the GI motility in vitro and in vivo, in physiological and pathophysiological conditions. Since abdominal pain is one of the most common symptoms of IBS, we also used animal models to evaluate the antinociceptive effects of *C. zacatechichi* extracts. The possible CNS-related effects of the extracts were evaluated by measurement of their effect on locomotor activity of mice. The chemical composition of the most active extract was evaluated by mass spectrometry to characterize the potential active compounds.

## Materials and methods

### Plant material

Dried shredded herb of *C*. *zacatechichi* (leaves, stems, and flowers) was purchased from the company Maya Ethnobotanicals (Haarlem, Netherlands). The material originated from Mexico according to the provider declaration. The authenticity of the purchased material was confirmed through the macroscopic and microscopic assessment which was carried out in comparison to the authenticated *C. zacatechichi* material from Daniel Siebert, provided by the School of Pharmacy, University of Mississippi. The botanical name conforms to the International Plant Names Index (Id: 187,802-1; Version: 1.2.2.1.1.3).

### Extracts preparation

Powdered *C. zacatechichi* herb (150 g) was extracted four times with boiling water. The aqueous solution was filtered and lyophilized (water fraction). This extraction was carried out due to the fact that in ethnomedicine, *C. zacatechichi* is employed against GI disorders in this form (Mayagoitia et al. 1986). However, in our preliminary studies, the aqueous extract did not exhibit any biological activity; thus, we did not use it in subsequent experiments (data not shown). The plant residue was dried in a dryer at 50 °C for 1 day and extracted with methanol. Methanolic extract was filtered and evaporated giving solid fraction (methanolic fraction). The residue was extracted with dichloromethane (DCM). The solution was evaporated, and as a result, 1.5 g of solid extract was obtained.

### Animals

Experimentally naive male C57BL/6N mice were obtained from the Animal House of the University of Lodz, Poland. All animals (7–8 weeks old) used in experiments weighed 22–30 g. Mice were housed at a constant temperature (22 °C) and maintained under a 12-h light/dark cycle (lights on 6:00 a.m.) in sawdust-lined plastic cages with access to chow pellets and tap water ad libitum. All animal protocols were in accordance with the European Communities Council Directive of 24 November 1986 (86/609/EEC) and Polish legislation acts concerning animal experimentation. The experimental protocol was approved by the Local Ethics Committee at the Medical University of Lodz (#590/2012). All efforts were made to minimize animal suffering and to reduce the number of animals used. All experiments conducted in these study are based on the well-established methodology which is routinely used in the authors’ laboratory (for reference please see Zielinska et al. (2015), Fichna et al. (2014a, b), Sałaga et al. (2014) and Fichna et al. (2014a, b)) and produce repetitive results. Hence, we did not use positive controls in all experiments to validate the models. We used loperamide as a positive control in the model of diarrhea for the sake of comparison of its effect with the antidiarrheal effect of the *C. zacatechichi* DCM extract.

### In vitro experiments on isolated smooth muscle strips

Organ bath experiments were performed according to the method described elsewhere (Sałaga et al. 2014). Briefly, mice were sacrificed by cervical dislocation. Full-thickness segments (1 cm) of distal colon were removed and kept in ice-cold oxygenated Krebs solution (NaCl 115 mM, KCl 8.0 mM, KH_2_PO_4_ 2.0 mM, NaHCO_3_ 25 mM, MgCl_2_ 2.4 mM, CaCl_2_ 1.3 mM, and glucose 10 mM). All experiments lasted less than 3 h, and each preparation was used for a single experiment only. Each segment was mounted between two platinum electrodes in organ baths containing Krebs (25 ml) equilibrated with 95 % O_2_ and 5 % CO_2_ at 37 °C. One end of each preparation was attached to the bottom of the organ bath using a silk thread, while the other end was connected to a FT03 force displacement transducer (Grass Technologies, West Warwick, RI, USA). Tension (0.5 g) was applied, and the preparations were allowed to equilibrate for 30 min. Changes in tension were amplified by a P11T amplifier (Grass Technologies, West Warwick, RI, USA) and recorded on a personal computer using the POLYVIEW software (Polybytes Inc., Cedar Rapids, IA, USA). Tissue strips were subjected to electrical field stimulation (EFS) applied by a S88X stimulator (Grass Technologies, EFS 8 Hz, 60 V, pulse duration 0.5 ms, train duration 10 s). EFS of isolated smooth muscle strips caused twitch contractions, which were virtually abolished by the muscarinic receptor antagonist atropine (10^−6^ M) or the neural blocker tetrodotoxin (10^−6^ M).

The contractile responses were characterized in the presence of increasing cumulative concentrations of *C. zacatechichi* methanol and DCM extracts (both 10^–4.6^ to 10^–1.6^ mg/ml), with the contact time for each concentration of 10 min. Before the addition of drugs, the mean amplitude of four successive twitch contractions was used as an internal control. Changes in contractions were reported as the percentage of the internal control. In control experiments, the effect of the vehicle was tested.

### In vivo investigation of whole gastrointestinal transit

Whole gut transit test evaluates the time of passage of the nonabsorbable colored marker (150 μl of glutinous liquid, consisting of 5 % Evans blue and 5 % Arabic gum) through the GI tract. Vehicle, *C. zacatechichi* methanol, or DCM extracts (200 mg/kg, p.o.) were injected 15 min before marker administration. Immediately after the intragastric administration of the marker, mice were returned to individual cages, which were placed on a white sheet in order to facilitate recognition of colored boluses. Time elapsed between intragastric administration of the marker and the excretion of the first colored fecal bolus was considered as whole gut transit time.

### Colonic bead expulsion test

Distal colonic expulsion was measured as reported recently (Sibaev et al. 2009). Briefly, animals were fasted overnight; *C*. *zacatechichi* DCM extract (100, 200, and 300 mg/kg, p.o.) or vehicle were administered p.o. (150 μL), and 15 min later, a prewarmed (37 °C) glass bead (2-mm diameter) was inserted into the distal colon (2-cm depth) using a silicone pusher. After insertion of the bead, mice were moved to individual cages and the time to bead expulsion was measured. Mice that did not expel the bead within 30 min were sacrificed to confirm the presence of the bead in the lumen of the colon.

### Mouse model of castor oil-induced diarrhea

To induce diarrhea, animals (fasted for 12 h before experiment) were gavaged with 200 μl of castor oil and then placed into individual cages for observation. Clean paper was put under each cage to improve the contrast. The time elapsed between administration of castor oil and the appearance of first symptoms of diarrhea (excretion of liquid feces) was measured and compared between groups. Loperamide (LOP) was used as a reference drug. *C. zacatechichi* DCM extract (200 mg/kg, p.o.), LOP (1 mg/kg, intraperitoneal; i.p.), or vehicle (p.o.) was administered 15 min prior to the gavage of the castor oil. The methodology was described earlier by Sałaga et al. (2014).

### Behavioral pain responses

Behavioral responses to intracolonic (i.c.) administration of mustard oil (MO, allyl isothiocyanate) were determined as described previously (Laird et al 2001; Eijkelkamp et al. 2007). Briefly, Vaseline was applied to the perianal area to exclude the stimulation of somatic areas and then 50 μl of MO (1 % in 70 % ethanol) was injected i.c. under isoflurane anesthesia. After 5 min of recovery, spontaneous behaviors were recorded on a videotape for 20 min for later analysis by an observer blinded to experimental conditions. Pain-related behaviors including (1) licking of the abdomen, (2) squashing of lower abdomen against the floor, (3) stretching the abdomen, and (4) abdominal retractions were each counted as 1.

*C. zacatechichi* DCM extract (200 mg/kg, p.o.) was administered 15 min before the MO instillation. Five percent dimethyl sulfoxide in saline was used as vehicle in control experiments.

The writhing test was performed as described earlier (Laird et al. 2001; Gach et al. 2010; Fichna et al. 2013). Fifteen minutes after administration of vehicle or *C. zacatechichi* DCM extract (200 mg/kg, p.o.), mice were injected i.p. with acetic acid solution (10 ml/kg of 0.75 %, *v*/*v* in saline). After the injection, mice were placed in individual cages for observation and the total number of writhes was counted 5 min later, during three periods of 5 min each. The writhing response, regarded as a nociceptive behavior, was characterized by elongation of the body and the development of tension in the abdominal muscles and hind paws.

### Measurement of locomotor activity

Locomotor activity was assessed according to the method described by Cravezic et al. (2011). Briefly, measurement was made automatically in a Digiscan actimeter (Omnitech Electronics Inc., Columbus, OH, USA), which monitored horizontal displacements and vertical movements. The animals were placed individually in 20 × 20 × 30 cm compartments, in a dimly illuminated and quiet room. The responses were expressed as the number of crossed infrared beams by mouse during four consecutive 15-min periods.

### LC-MS analysis of *C. zacatechichi* DCM extract

A tandem mass spectrometer MicrOTOF-Q II from Bruker Daltonik (Bremen, Germany) coupled with an LC-UV Ultimate 3000 system (Dionex, a part of Thermo Fisher Scientific) was used to obtain the electrospray ionization time-of-flight mass spectra (LC-ESI-MS/MS-TOF). The following settings were used: electrospray ionization (ESI) in the positive ion mode, dry gas (nitrogen) flow rate 8.0 l min^−1^, the dry heater 180 °C, the capillary voltage 4500 V, and end plate offset −500 V. MS data were recorded in the full scan mode (from 50 to 3000 *m*/*z*). Data processing was carried out with Compass 1.3 (Bruker Daltonik).

Chromatographic analysis was performed on a C_18_ analytical column (Hypersil GOLD, 150 mm × 2.1 mm; 3-μm particle size; Thermo Fisher Scientific, Waltham, MA, USA) with a guard column (Hypersil GOLD, 10 mm × 2.1 mm; 3-μm particle size; Thermo Fisher Scientific). The linear gradient elution was performed using 0.1 % formic acid in solvent A (water/acetonitrile, 9:1, *v*/*v*) and 0.1 % formic acid in solvent B (methanol/acetonitrile, 9:1, *v*/*v*). An applied linear gradient was as follows: initially 10 % B from 0 to 2 min, then linear gradient to 90 % B at 7 min, constant 90 % B to 10 min, and finally return to 10 % B and equilibration for 2 min. The flow rate was 0.15 ml min^−1^, and the injection volume was 2 μl. The analysis was carried out at 25 °C.

The identification of individual compounds was based on the analysis of high resolution mass spectra. Time of flight analyzer enables a very accurate mass detection, resulting in the assignment to the most probable molecular formula. Suggested molecular formulas were accepted when the mass error was below 5 ppm. The identification was further enhanced by the analysis of the isotopic pattern of the compounds. If possible, obtained MS/MS spectra were additionally compared with spectra of identified compounds in the METLIN: Metabolite and Tandem MS Database (http://metlin.scripps.edu).

### Drugs

All drugs and reagents, unless otherwise stated, were purchased from Sigma-Aldrich (Poznan, Poland). Loperamide was purchased from Tocris Bioscience (Bristol, UK). In the in vitro experiments (isolated smooth muscle strips), all drugs were dissolved in dimethyl sulfoxide. In the in vivo tests, drugs were dissolved in 5 % dimethyl sulfoxide in saline, which was used as vehicle in control experiments. The vehicles in the used concentrations had no effects on the observed parameters.

### Statistics

In the in vitro experiments, *n* denotes the number of individual tissues from at least three different animals. Statistical analyses were performed using PRISM 5.0 (GraphPad Software Inc., La Jolla, CA, USA). The data are expressed as means ± SEM. Student’s *t* test was used to compare single treatment means with control means. Analysis of variance (ANOVA) followed by Newman-Keuls post hoc test was used for analysis of multiple treatment means. *P* values ≤0.05 were considered statistically significant.

## Results

### Inhibitory activity of *C. zacatechichi* on ex vivo smooth muscle contractility

**Fig. 1:**
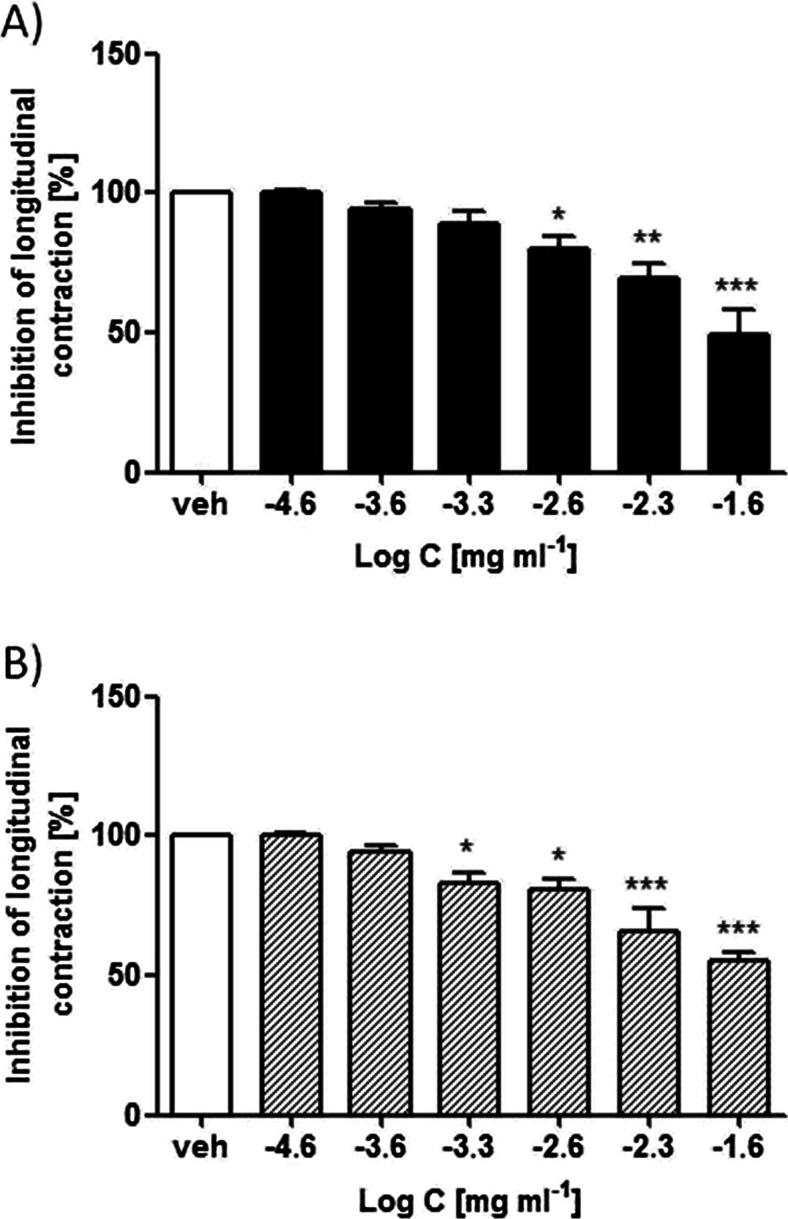
Effect of *C. zacatechichi* DCM (**a**) and methanol (**b**) extracts (10^–4.6^–10^–1.6^ mg/ml) on EFS-stimulated longitudinal smooth muscle contractions in mouse colon (8 Hz, 60 V, pulse duration 0.5 ms, train duration 10 s). Data represent mean ± SEM for *n* = 4–7 experiments. *P < 0.05, **P < 0.01, ***P < 0.001 as compared to control

We first investigated the effect of *C. zacatechichi* methanol and DCM extracts on isolated mouse colon contractility. Both extracts (10^–4.6^–10^–1.6^ mg/ml) inhibited the EFS-induced twitch contraction to a comparable extent, in a concentration-dependent manner (IC_50_ value of 16.66 ± 1.54 μg/ml and 18.48 ± 1.22 μg/ml for DCM and methanol extracts, respectively, Fig. 1a, b). The maximum inhibitory effect of both compounds was approximately 50 %.

### In vivo inhibitory activity of *C. zacatechichi* in whole gastrointestinal transit test

**Fig. 2:**
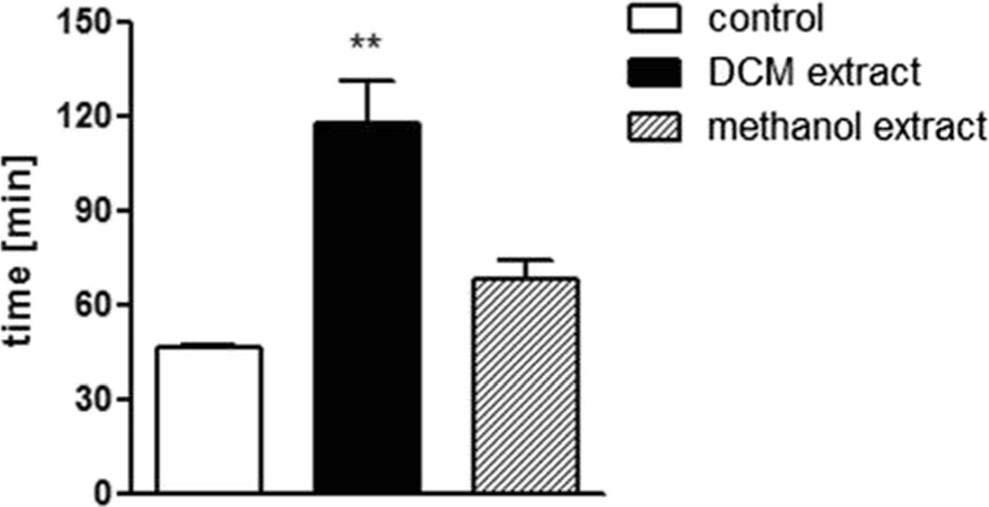
Effect of *C. zacatechichi* DCM and methanol extracts (both 200 mg/kg, p.o.) on whole GI transit time. Data represent mean ± SEM of *n* = 6–10 animals. **P < 0.01, as compared with the control group

As shown in Fig. 2, only DCM extract, administered p.o., produced a potent, inhibitory effect on whole GI motility. Therefore, the DCM extract was used in subsequent experiments.

### In vivo inhibitory activity of *C. zacatechichi* colonic bead expulsion test

**Fig. 3:**
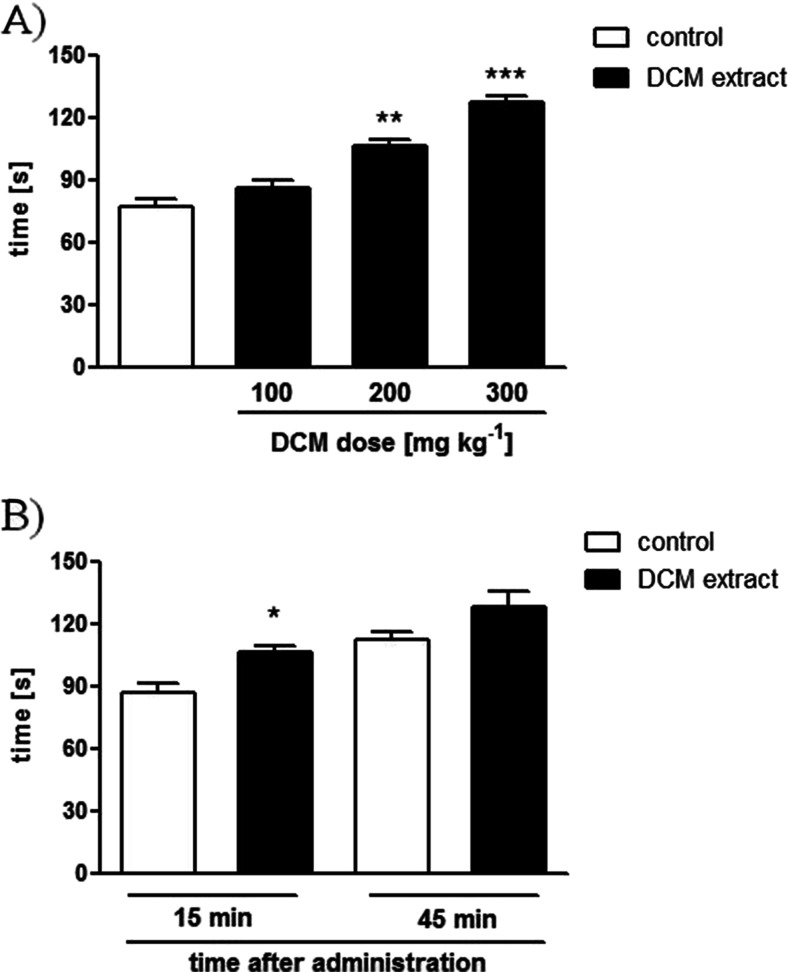
Effect of *C. zacatechichi* DCM extract on lower GI tract motility in the colonic bead expulsion test. The dose-effect relationship of 100, 200, and 300 mg/kg, p.o. on the colonic bead expulsion time (**a**). Time course of changes of bead expulsion time after administration of *C. zacatechichi* DCM extract at the dose of 200 mg/kg, p.o. (**b**). Two control groups (15 and 45 min after administration of the DCM extract) were used to take into account the adaptive changes in the colonic smooth muscle that typically occur after the second insertion of the bead. Data represent mean ± SEM of *n* = 6–8 mice for each experimental group. *P < 0.05, **P < 0.01, ***P < 0.001, as compared with control

*C. zacatechichi* DCM extract produced a significant dose-dependent inhibitory effect on colonic expulsion 15 min after administration (Fig. 3a). The effect was relatively short lasting, as it was not observed 45 min after administration of the extract (Fig. 3b).

This experiment showed that the oral dose of 200 mg/kg is sufficient to elicit significant effect on the GI tract motility in vivo. Hence, we proceeded with this dose in all subsequent in vivo experiments.

### Antidiarrheal activity of *C. zacatechichi* DCM extract in mouse

**Fig. 4:**
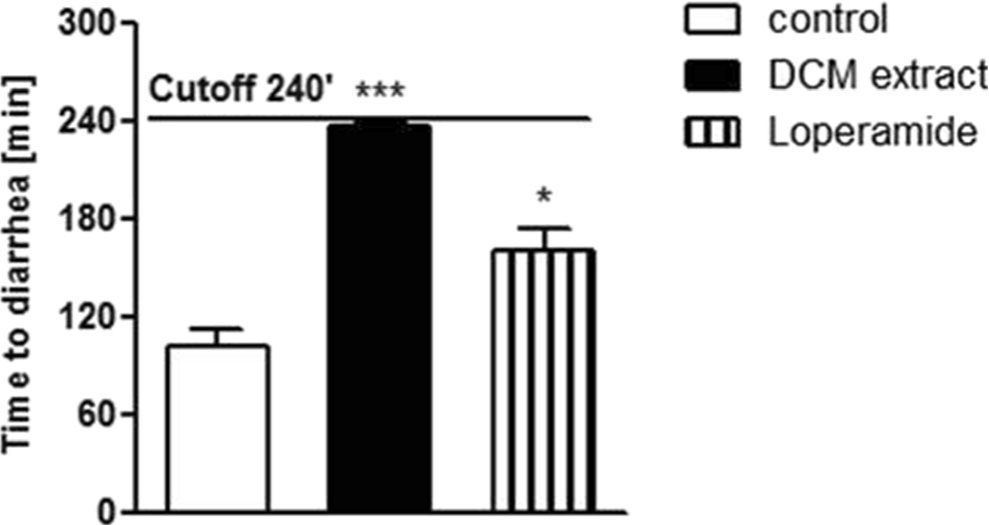
Antidiarrheal activity of *C*. *zacatechichi* DCM extract. The effect of *C*. *zacatechichi* DCM extract (200 mg/kg, p.o.) and LOP (1 mg/kg, i.p.) on the delay of the emergence of castor oil-induced diarrhea. Data represent mean ± SEM of *n* = 6–8 mice for each experimental group. *P < 0.05, ***P < 0.001, as compared to castor oil-treated/control animals

To investigate the antidiarrheal activity of *C. zacatechichi* DCM extract, we used a mouse model of castor oil-induced diarrhea. Intragastric administration of castor oil caused an accumulation of water and electrolytes in the mouse intestine, what resulted in an acute diarrhea in control animals (Fig. 4). *C. zacatechichi* DCM extract (200 mg/kg p.o.) significantly delayed the emergence of liquid feces, and its effect was more marked than that obtained with the antidiarrheal drug loperamide (1 mg/kg, i.p.); more specifically, the extract prevented diarrhea in nearly all experimental animals until the cutoff time (Fig. 4).

### Antinociceptive activity of *C. zacatechichi* DCM extract in mouse models of abdominal pain

**Fig. 5:**
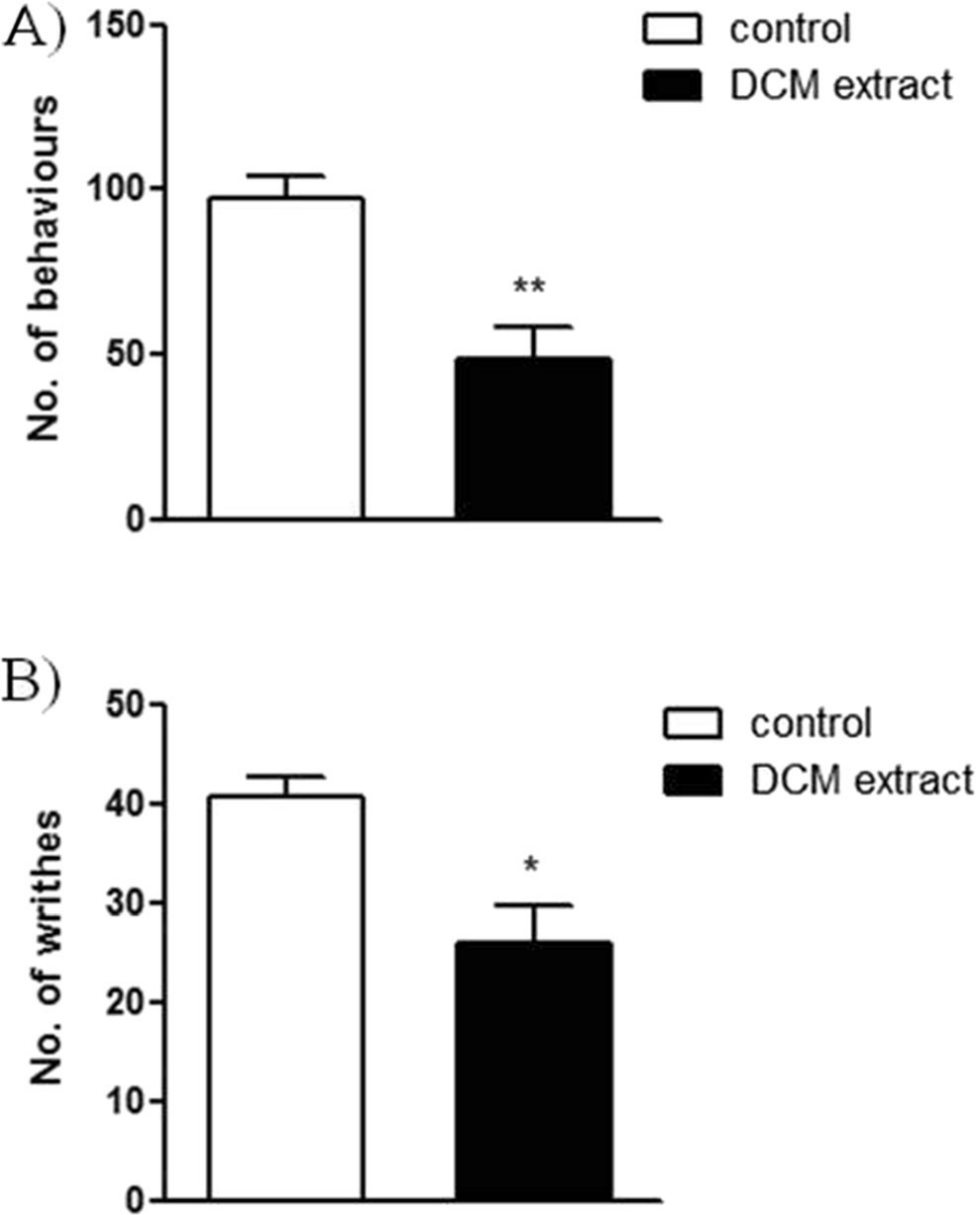
Antinociceptive activity of *C*. *zacatechichi* DCM extract. **a** The effect of *C. zacatechichi* DCM extract (200 mg/kg, p.o.) on the number of pain-related behaviors evoked by i.c. administration of mustard oil (MO). **b** The effect of p.o. administered *C. zacatechichi* DCM extract (200 mg/kg) on the number of writhes. Data represent mean ± SEM of *n* = 6–8 mice per group. *P < 0.05, **P < 0.01, as compared to MO/acetic acid-treated animals

In order to assess the antinociceptive activity of *C. zacatechichi* DCM extract, two different mouse models of abdominal pain were used. In the behavioral model elicited by the i.c. injection of MO, the p.o. administration of *C. zacatechichi* DCM extract (200 mg/kg) resulted in a significant antinociceptive effect (Fig. 5a). Similarly, the p.o. administration of *C. zacatechichi* DCM extract at the dose of 200 mg/kg resulted in a significant reduction of the number of writhes (Fig. 5b).

### *C. zacatechichi* DCM extract does not affect motor functions in mice

**Fig. 6:**
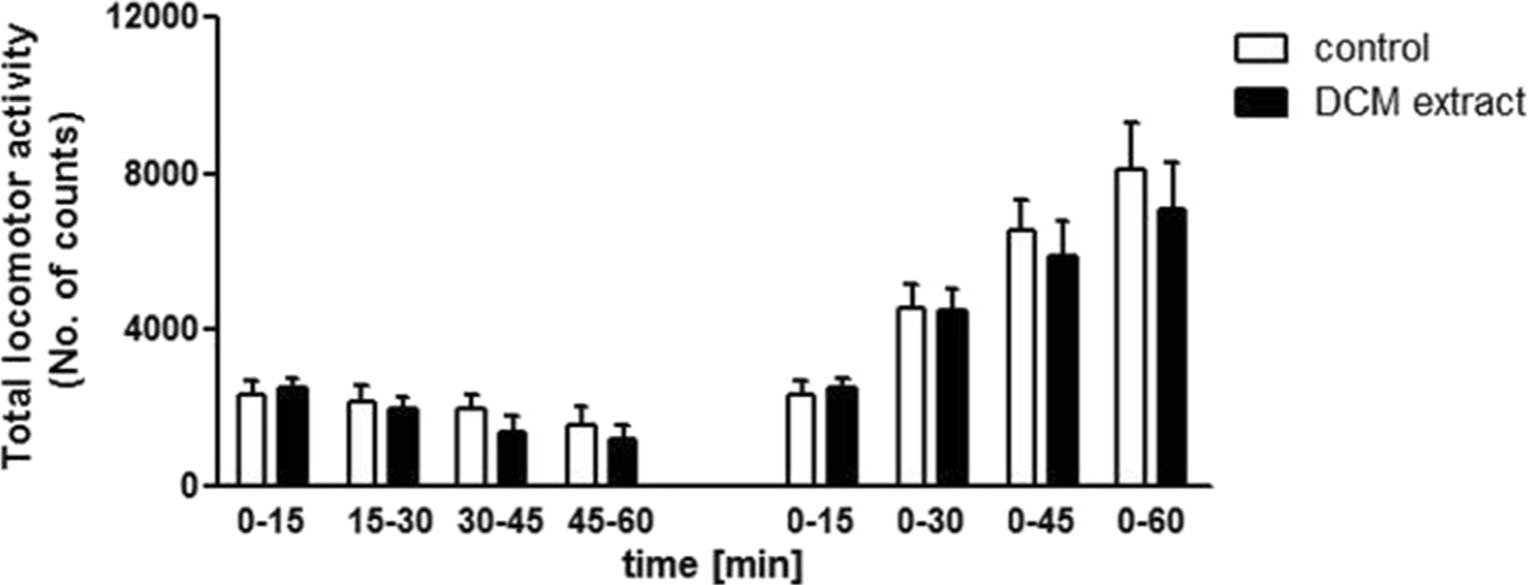
Effect of *C. zacatechichi* DCM extract (200 mg/kg, p.o.) on total locomotor activity. *Bars on the left side* of the graph represent total number of counts in subsequent 15-min time period. *Bars on the right side* of the graph show cumulative values of a total number of counts in the time periods indicated on the OX axis of the graph. Depicted are means ± SEM of *n* = 6–8 mice per group

The influence of the p.o. administration of *C. zacatechichi* DCM extract on mouse locomotor activity was measured over six consecutive periods of 10 min each. The extract administered at a dose of 200 mg/kg did not modify total locomotor activity in any of the time periods of the test (Fig. 6).

### The LC-MS analysis of *C. zacatechichi* DCM extract

Table 1 reports the measured accurate ion mass (M+H)^+^ or (M+Na)^+^ of the analyzed compounds and the corresponding most probable molecular formula. The liquid chromatography-mass spectrometry (LC-MS) analysis of the extract showed the presence of nine constituents, which are coumarin derivatives (1, 2, 3), flavone derivative (4), and germacranolides (5, 6, 7, 8, 9; Table 1). Comparison of the mass spectra of the constituents with the METLIN library or published papers (Chouchi and Barth 1994; Wu et al. 2011; Liu et al. 2012) resulted in the following list of compounds: 7-methoxy-2*H*-chromen-2-one (1); 5,7-dimethoxy-2*H*-chromen-2-one (2); 6,7,8-trimethoxy-2*H*-chromen-2-one (3); acacetin (4); calealactones: C (6), D (9), and E (5); 8β-angeloxy-9α-acetyloxycalyculatolide (7); and calein A (8).

**Table 1:** Chemical characterization of the *C. zacatechichi* DCM extract

No.	^a^[M + H]^+^ ^b^[M + Na]^+^ (*m*/*z*)	Empirical formula	Name	Reference
1	^a^177.056	C_10_H_8_O_3_	7-Methoxy-2*H*-chromen-2-one	Kielbus et al. 2013
2	^a^207.066	C_11_H_10_O_4_	5,7-Dimethoxy-2*H*-chromen-2-one	Chouchi and Barth 1994
3	^a^237.077	C_12_H_12_O_5_	6,7,8-Trimethoxy-2*H*-chromen-2-one	Galabov et al. 1996
4	^a^285.077	C_16_H_12_O_5_	Acacetin	Liu et al. 2012
5	^a^363.180	C_20_H_26_O_6_	Calealactone E	Wu et al. 2011
6	^a^407.170	C_21_H_26_O_8_	Calealactone C	Wu et al. 2011
7	^a^419.170	C_22_H_26_O_8_	8β-angeloxy-9α-acetyloxycalyculatolide	Wu et al. 2011
8	^a^421.185	C_22_H_28_O_8_	Calein A	Wu et al. 2011
9	^b^443.170	C_22_H_28_O_8_	Calealactone D (sodium adduct)	Wu et al. 2011

The superscript letters before the values correspond to the superscript letters in the header

## Discussion

In this study, we characterized the effect of *C. zacatechichi* DCM extract on motility and abdominal pain with in vitro and in vivo tests. We found that the extract is a strong regulator of intestinal motility in both physiological and pathophysiological conditions as well as pain signaling. The latter effect is particularly important since abdominal pain is one of the major symptoms of all types of IBS.

Plant extracts are a well-known source of biologically active compounds, which may be used for the treatment of various conditions, e.g., IBS, in which classical pharmacological therapies do not provide sufficient relief. Depending on the main symptom, three types of IBS may be distinguished: constipation-predominant (IBS-C), diarrhea-predominant (IBS-D), and altering bowel habits (IBS-A). The major goals of anti-IBS therapies are control of colonic motility and abdominal pain. Currently available pharmacological treatments are mainly targeted at reduction of symptoms but not complete healing. In addition, some patients may have coexisting conditions that contribute to the severity of IBS symptoms, requiring further consideration when choosing treatment options (Yoon et al. 2011).

To date, various classes of drugs have been tested as potential anti-IBS therapy, for instance 5-HT_3_ antagonists and bile acid-binding agents (for IBS-D), 5-HT_4_ agonists, and chloride channel activators (for IBS-C) (Manabe et al. 2010). Moreover, peripheral kappa-opioid receptor agonists such as asimadoline or salvinorin A derivatives have been proposed as visceral analgesics in IBS-D (Manabe al. 2010; Sałaga et al. 2014). However, many of these drugs do not improve sufficiently disease symptoms or may cause side effects which force patients to seek nonprescribed pharmacological regimens falling within complementary and alternative medicine (CAM) therapies. A study by Kong et al. (2005) has shown that the incidence of CAM use is 50.9 % for IBS patients; moreover, about 50 % of CAM users perceived improvement of symptoms (Yoon et al. 2011).

Several studies concerning the use of single herbal medicines in IBS have recently been reported. For instance, it has been shown that 4-week treatment with enteric-coated peppermint capsules twice daily or before meals significantly reduces IBS symptoms and increases quality of life (QOL) (Cappello et al. 2007; Merat et al. 2010). Moreover, Bundy et al. (2004a and 2004b) have reported the anti-IBS activity of turmeric and artichoke extracts, which significantly improved the IBS symptoms and QOL. Finally, multiple herbal preparations such as Iberogast, Padma Lax, and Tong Xie Yao Fang have been tested for anti-IBS activity and are described in detail by Yoon et al. (2011). These data indicate that natural product-derived preparations constitute an important branch of anti-IBS medications.

Here, for the first time, we report the actions of *C. zacatechichi* DCM extract, which may be employed in the treatment of IBS. We found that the extract inhibited motility in the lower GI tract in a dose-dependent manner. This is particularly important in the treatment of IBS-D, where disrupted colonic motility enhances the frequency of defecation. Moreover, in pathophysiological conditions, *C. zacatechichi* DCM extract exhibited outstanding antidiarrheal activity, even more potent than that of a well-known drug loperamide, and was active after oral administration. Altered intestinal motility is often accompanied with abdominal pain, which is one of the most common symptoms that lead IBS patients to gastroenterology clinics. Consequently, in this study, we showed that *C. zacatechichi* DCM extract alleviates abdominal pain in two well-established animal models after oral administration. These features make the extract a promising candidate for a source of anti-IBS compounds.

Chemical analysis of the *C. zacatechichi* DCM extract resulted in the identification of several germacranolides, which were previously detected by Wu et al. (2011). These compounds were shown to have antileishmanial and antimicrobial activities in vitro. Moreover, Umemura et al. (2008) demonstrated that germacranolides such as calealactone A activate the nuclear factor E2-related factor 2 (Nrf2)/antioxidant response element (ARE) pathway and induce the phase II detoxification/antioxidant enzymes upon oxidative stress, thereby resulting in an increased resistance to oxidative damage. The next group of compounds which we identified in DCM extract of *C. zacatechichi* was coumarin derivatives. Coumarin is a well-known anticoagulant, and to date, it was not shown to exhibit antidiarrheal activity. On the other hand, recent data suggest that coumarin as well as its derivatives produce antinociceptive effect in the writhing test in mice after oral administration (Park et al. 2013; Alipour et al. 2014). This experimental data suggest the contribution of coumarin derivatives in inhibition of abdominal pain in our study. We also detected the presence of acacetin in our extract. However, we did not find any literature data concerning the effect of this compound on the GI tract.

As known from occasional users as well as one scientific report, *C. zacatechichi* may affect the CNS. At the doses used by humans, organic extracts of the plant produce behavioral signs of somnolence and induce light sleep in cats (Mayagoitia et al. 1986). Large doses may produce other side effects, such as salivation, ataxia, retching, and occasional vomiting. A controlled nap sleep study showed that *C. zacatechichi* extracts increased the number of superficial stages of sleep and the number of spontaneous awakenings (Mayagoitia et al. 1986). Moreover, Mayagoitia et al. (1986) demonstrated that methanol extract elicits larger psychopharmacologic effects than hexane extract, suggesting that active compounds might be found in the polar rather than nonpolar fraction. Taking that into consideration, one of our most prominent goals was to evaluate whether nonpolar *C. zacatechichi* DCM extract produces CNS-related effects. We found that the extract did not affect locomotor activity of animals when administered at the doses which produce potent antidiarrheal and antinociceptive effects in the GI tract. We thus suggest that psychoactive constituents of *C. zacatechichi* are not present in DCM extract, what guarantees potential safe use of the preparation in patients. However, further studies are warranted to confirm this observation.

## Conclusion

*C. zacatechichi* DCM extract exhibits potent action in the GI tract and produces a significant antidiarrheal and antinociceptive effect in mouse models mimicking IBS-D. Moreover, our data suggest that the preparation does not elicit behavioral changes associated with CNS-related effects of *C. zacatechichi.* Furthermore, the extract is active after oral administration. Further chemical analyses of *C. zacatechichi* are thus urgently needed in order to find all its active constituents, which may open up new opportunities for the discovery of novel therapeutics for functional GI diseases, including IBS-D.

## References

[CR1] Alipour M, Khoobi M, Emami S, Fallah-Benakohal S, Ghasemi-Niri SF, Abdollahi M, Foroumadi A, Shafiee A (2014). Antinociceptive properties of new coumarin derivatives bearing substituted 3,4-dihydro-2H-benzothiazines. Daru.

[CR2] Atkinson W, Sheldon TA, Shaath N, Whorwell PJ (2004). Food elimination based on IgG antibodies in irritable bowel syndrome: a randomised controlled trial. Gut.

[CR3] Bork PM, Schmitz ML, Kuhnt M, Escher C, Heinrich M (1997). Sesquiterpene lactone containing Mexican Indian medicinal plants and pure sesquiterpene lactones as potent inhibitors of transcription factor NF-kappaB. FEBS Lett.

[CR4] Bundy R, Walker AF, Middleton RW, Booth J (2004). Turmeric extract may improve irritable bowel syndrome symptomology in otherwise healthy adults: a pilot study. J Altern Complement Med.

[CR5] Bundy R, Walker AF, Middleton RW, Marakis G, Booth JC (2004). Artichoke leaf extract reduces symptoms of irritable bowel syndrome and improves quality of life in otherwise healthy volunteers suffering from concomitant dyspepsia: a subset analysis. J Altern Complement Med.

[CR6] Cappello G, Spezzaferro M, Grossi L, Manzoli L, Marzio L (2007). Peppermint oil (Mintoil) in the treatment of irritable bowel syndrome: a prospective double blind placebo-controlled randomized trial. Dig Liver Dis.

[CR7] Chouchi D, Barth D (1994). Rapid identification of some coumarin derivatives in deterpenated citrus peel oil by gas chromatography. J Chromatogr A.

[CR8] Cravezic A, Fichna J, Gach K, Wyrebska A, Perlikowska R, Costentin J, Bonnet JJ, Janecka A, Do Rego JC (2011). Effect of potent endomorphin degradation blockers on analgesic and antidepressant-like responses in mice. Neuropharmacology.

[CR9] Eijkelkamp N, Kavelaars A, Elsenbruch S, Schedlowski M, Holtmann G, Heijnen CJ (2007). Increased visceral sensitivity to capsaicin after DSS-induced colitis in mice: spinal cord c-Fos expression and behavior. Am J Physiol Gastrointest Liver Physiol.

[CR10] Fichna J, Storr MA (2012). Brain-gut interactions in IBS. Front Pharmacol.

[CR11] Fichna J, Sibaev A, Salaga M, Sobczak M, Storr M (2013). The cannabinoid-1 receptor inverse agonist taranabant reduces abdominal pain and increases intestinal transit in mice. Neurogastroenterol Motil.

[CR12] Fichna J, Sobczak M, Mokrowiecka A, Cygankiewicz AI, Zakrzewski PK, Cenac N, Sałaga M, Timmermans JP, Vergnolle N, Małecka-Panas E, Krajewska WM, Storr M (2014). Activation of the endogenous nociceptin system by selective nociceptin receptor agonist SCH 221510 produces antitransit and antinociceptive effect: a novel strategy for treatment of diarrhea-predominant IBS. Neurogastroenterol Motil.

[CR13] Fichna J, Sałaga M, Stuart J, Saur D, Sobczak M, Zatorski H, Timmermans JP, Bradshaw HB, Ahn K, Storr MA (2014). Selective inhibition of FAAH produces antidiarrheal and antinociceptive effect mediated by endocannabinoids and cannabinoid-like fatty acid amides. Neurogastroenterol Motil.

[CR14] Gach K, do-Rego JC, Fichna J, Storr M, Delbro D, Toth G, Janecka A. (2010). Synthesis and biological evaluation of novel peripherally active morphiceptin analogs. Peptides 31: 1617-1624 10.1016/j.peptides.2010.04.018 20434497

[CR15] Galabov AS, Iosifova T, Vassileva E, Kostova I (1996). Antiviral activity of some hydroxycoumarin derivatives. Z Naturforsch C.

[CR16] Kielbus M, Skalicka-Wozniak K, Grabarska A, Jeleniewicz W, Dmoszynska-Graniczka M, Marston A, Polberg K, Gawda P, Klatka J, Stepulak A (2013). 7-substituted coumarins inhibit proliferation and migration of laryngeal cancer cells in vitro. Anticancer Res.

[CR17] Kong SC, Hurlstone DP, Pocock CY, Walkington LA, Farquharson NR, Bramble MG, McAlindon ME, Sanders DS (2005). The incidence of self-prescribed oral complementary and alternative medicine use by patients with gastrointestinal diseases. J Clin Gastroenterol.

[CR18] Laird JM, Martinez-Caro L, Garcia-Nicas E, Cervero F (2001). A new model of visceral pain and referred hyperalgesia in the mouse. Pain.

[CR19] Leonti M, Sticher O, Heinrich M (2003). Antiquity of medicinal plant usage in two Macro-Mayan ethnic groups (Mexico). J Ethnopharmacol.

[CR20] Liu J, Chen L, Cai S, Wang Q (2012). Semisynthesis of apigenin and acacetin-7-O-beta-D-glycosides from naringin and their cytotoxic activities. Carbohydr Res.

[CR21] Manabe N, Rao AS, Wong BS, Camilleri M (2010). Emerging pharmacologic therapies for irritable bowel syndrome. Curr Gastroenterol Rep.

[CR22] Mayagoitia L, Diaz JL, Contreras CM (1986). Psychopharmacologic analysis of an alleged oneirogenic plant: Calea zacatechichi. J Ethnopharmacol.

[CR23] Mayer EA, Collins SM (2002). Evolving pathophysiologic models of functional gastrointestinal disorders. Gastroenterology.

[CR24] Merat S, Khalili S, Mostajabi P, Ghorbani A, Ansari R, Malekzadeh R (2010). The effect of enteric-coated, delayed-release peppermint oil on irritable bowel syndrome. Dig Dis Sci.

[CR25] Park SH, Sim YB, Kang YJ, Kim SS, Kim CH, Kim SJ, Lim SM, Suh HW (2013). Antinociceptive profiles and mechanisms of orally administered coumarin in mice. Biol Pharm Bull.

[CR26] Philpott H, Gibson P, Thien F (2011). Irritable bowel syndrome—an inflammatory disease involving mast cells. Asia Pac Allergy.

[CR27] Rahimi R, Abdollahi M (2012). Herbal medicines for the management of irritable bowel syndrome: a comprehensive review. World J Gastroenterol.

[CR28] Sałaga M, Polepally PR, Sobczak M, Grzywacz D, Kamysz W, Sibaev A, Storr M, Do Rego JC, Zjawiony JK, Fichna J (2014). Novel orally available salvinorin A analog PR-38 inhibits gastrointestinal motility and reduces abdominal pain in mouse models mimicking irritable bowel syndrome. J Pharmacol Exp Ther.

[CR29] Sandler RS, Everhart JE, Donowitz M, Adams E, Cronin K, Goodman C, Gemmen E, Shah S, Avdic A, Rubin R (2002). The burden of selected digestive diseases in the United States. Gastroenterology.

[CR30] Sibaev A, Yuce B, Kemmer M, Van NL, Broedl U, Allescher HD, Goke B, Timmermans JP, Storr M (2009). Cannabinoid-1 (CB1) receptors regulate colonic propulsion by acting at motor neurons within the ascending motor pathways in mouse colon. Am J Physiol Gastrointest Liver Physiol.

[CR31] Thompson WG, Heaton KW, Smyth GT, Smyth C (2000). Irritable bowel syndrome in general practice: prevalence, characteristics, and referral. Gut.

[CR32] Umemura K, Itoh T, Hamada N, Fujita Y, Akao Y, Nozawa Y, Matsuura N, Iinuma M, Ito M (2008). Preconditioning by sesquiterpene lactone enhances H2O2-induced Nrf2/ARE activation. Biochem Biophys Res Commun.

[CR33] Wu H, Fronczek FR, Burandt CL, Zjawiony JK (2011). Antileishmanial germacranolides from Calea zacatechichi. Planta Med.

[CR34] Yoon SL, Grundmann O, Koepp L, Farrell L (2011). Management of irritable bowel syndrome (IBS) in adults: conventional and complementary/alternative approaches. Altern Med Rev.

[CR35] Zielińska M, Chen C, Mokrowiecka A, Cygankiewicz AI, Zakrzewski PK, Sałaga M, Małecka-Panas E, Wlaź P, Krajewska WM, Fichna J (2015). (2015). Orally administered novel cyclic pentapeptide P-317 alleviates symptoms of diarrhoea-predominant irritable bowel syndrome.. J Pharm Pharmacol.

